# Bis[4-(2-isopropyl-2*H*-tetra­zol-5-yl)phen­yl]dimethyl­silane

**DOI:** 10.1107/S160053681100033X

**Published:** 2011-01-12

**Authors:** Peng Wang, Zheng Yue, Jie Zhang, Sheng-Yu Feng

**Affiliations:** a School of Chemistry and Chemical Engineering, Shandong University, 27 Shanda Nanlu Road, Jinan, People’s Republic of China

## Abstract

The title compound, C_22_H_28_N_8_Si, has crystallographic 2 symmetry with the Si atom located on a twofold rotation axis. The tetra­zole ring is oriented at a dihedral angle of 5.32 (18)° with respect to the benzene ring. A C—H⋯π inter­action occurs between adjacent mol­ecules in the crystal structure.

## Related literature

For applications of tetra­zole compounds, see: Bhandari *et al.* (2000 ▶). For the synthesis of tetra­zole derivatives, see: Demko & Sharpless (2001 ▶).
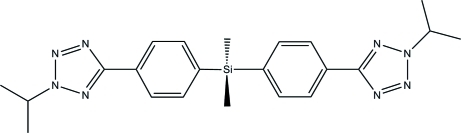


## Experimental

### 

#### Crystal data

C_22_H_28_N_8_Si
*M* *_r_* = 432.61
Orthorhombic, 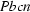

*a* = 7.2722 (14) Å
*b* = 11.536 (2) Å
*c* = 28.444 (6) Å
*V* = 2386.2 (8) Å^3^
*Z* = 4
Mo *K*α radiation
μ = 0.12 mm^−1^
*T* = 298 K
0.46 × 0.37 × 0.07 mm

#### Data collection

Bruker SMART CCD area-detector diffractometer
Absorption correction: multi-scan (*SADABS*; Sheldrick, 2004 ▶) *T*_min_ = 0.945, *T*_max_ = 0.991
12923 measured reflections
2613 independent reflections
1827 reflections with *I* > 2σ(*I*)
*R*_int_ = 0.049

#### Refinement

*R*[*F* ^2^ > 2σ(*F* ^2^)] = 0.088
*wR*(*F* ^2^) = 0.207
*S* = 1.14
2613 reflections
144 parameters
H-atom parameters constrained
Δρ_max_ = 0.32 e Å^−3^
Δρ_min_ = −0.19 e Å^−3^

### 

Data collection: *SMART* (Bruker, 2007 ▶); cell refinement: *SAINT* (Bruker, 2007 ▶); data reduction: *SAINT*; program(s) used to solve structure: *SHELXTL* (Sheldrick, 2008 ▶); program(s) used to refine structure: *SHELXTL*; molecular graphics: *SHELXTL*; software used to prepare material for publication: *SHELXTL*.

## Supplementary Material

Crystal structure: contains datablocks I, global. DOI: 10.1107/S160053681100033X/xu5118sup1.cif

Structure factors: contains datablocks I. DOI: 10.1107/S160053681100033X/xu5118Isup2.hkl

Additional supplementary materials:  crystallographic information; 3D view; checkCIF report

## Figures and Tables

**Table 1:** Hydrogen-bond geometry (Å, °) *Cg* is the centroid of the tetra­zole ring.

*D*—H⋯*A*	*D*—H	H⋯*A*	*D*⋯*A*	*D*—H⋯*A*
C9—H9*B*⋯*Cg*^i^	0.96	2.86	3.738 (5)	152

Symmetry code: (i) 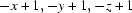.

**Fig. 1.**
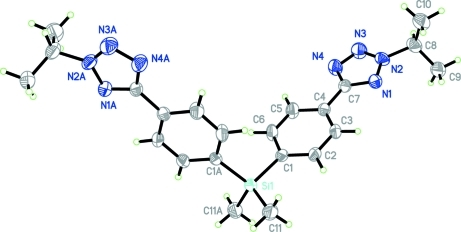
The molecular structure of (I), with atom labels and 30% probability displacement ellipsoids.

## supplementary crystallographic information

### Comment

Due to the application of tetrazoles in coordination chemistry, medicinal chemistry and material science (Bhandari *et al.*, 2000), series of organic compounds with tetrazole group have been synthesized through different methods. Since a safe, convenient and envrironmentally friendly procedure for the synthesis of 5-substituted 1*H*-tetrazoles in water was reported by Demko and Sharpless (2001), synthesis of such compounds has been developed rapidly. However, due to the difficult in synthesis, tetrazole functional silane was never reported to our best knowledge. Here, we reported the synthesis and crystal structure of the title compound(I), namely, bis(4-(2-isopropyl-2*H*-tetrazol- 5-yl)phenyl)dimethylsilane.

The molecular structure of (I) is shown in Fig. 1. Bond lengths and angles in (I) are normal. The phenyl and tetrazole rings are not coplanar, and the two rings twisted to each other at a dihedral angle of 5.32 (18)°. The crystal packing is stablized by C—H···π interaction (Table 1).

### Experimental

Tert-butyl lithium (4 mmol) in 3.08 ml n-pentane solution and 5-(4-bromophenyl)-2-isopropyl-2*H*-tetrazole (0.54 g, 2 mmol) were reacted at 195 K in 20 ml e ther. To the resulted solution was added dimethyldichlorosilane (0.13 g, 1 mmol), the solution was warmed slowly to room temperature and stirred overnight. Then the solution was filtered. The volatiles were removed from the resulting filtrate by vacuum distillation. The residue was purified by column chromatography using ethyl acetate/n-hexane as eluent to afford the pure compound. Single crystals of (I) suitable for X-ray analysis were obtained by slow evaporation methanol solvent.

### Refinement

The H atoms were positioned geometrically and refined using a riding model with C—H = 0.93–0.96 Å and *U*_iso_(H) = 1.5*U*_eq_(C) for methyl and 1.2*U*_eq_(C) for the others.

### Crystal data

**Table:**

C_22_H_28_N_8_Si	*F*(000) = 920
*M**_r_* = 432.61	*D*_x_ = 1.204 Mg m^−^^3^
Orthorhombic, *P**b**c**n*	Mo *K*α radiation, λ = 0.71073 Å
Hall symbol: -P 2n 2ab	Cell parameters from 1662 reflections
*a* = 7.2722 (14) Å	θ = 2.9–20.6°
*b* = 11.536 (2) Å	µ = 0.12 mm^−^^1^
*c* = 28.444 (6) Å	*T* = 298 K
*V* = 2386.2 (8) Å^3^	Plate, colourless
*Z* = 4	0.46 × 0.37 × 0.07 mm

### Data collection

**Table:**

Bruker SMART CCD area-detector diffractometer	2613 independent reflections
Radiation source: fine-focus sealed tube	1827 reflections with *I* > 2σ(*I*)
graphite	*R*_int_ = 0.049
φ and ω scans	θ_max_ = 27.0°, θ_min_ = 1.4°
Absorption correction: multi-scan (*SADABS*; Sheldrick, 2004)	*h* = −9→9
*T*_min_ = 0.945, *T*_max_ = 0.991	*k* = −14→10
12923 measured reflections	*l* = −36→36

### Refinement

**Table:**

Refinement on *F*^2^	Primary atom site location: structure-invariant direct methods
Least-squares matrix: full	Secondary atom site location: difference Fourier map
*R*[*F*^2^ > 2σ(*F*^2^)] = 0.088	Hydrogen site location: inferred from neighbouring sites
*wR*(*F*^2^) = 0.207	H-atom parameters constrained
*S* = 1.14	*w* = 1/[σ^2^(*F*_o_^2^) + (0.0693*P*)^2^ + 1.6746*P*] where *P* = (*F*_o_^2^ + 2*F*_c_^2^)/3
2613 reflections	(Δ/σ)_max_ = 0.001
144 parameters	Δρ_max_ = 0.32 e Å^−^^3^
0 restraints	Δρ_min_ = −0.19 e Å^−^^3^

### Special details

**Table:**

Geometry. All e.s.d.'s (except the e.s.d. in the dihedral angle between two l.s. planes) are estimated using the full covariance matrix. The cell e.s.d.'s are taken into account individually in the estimation of e.s.d.'s in distances, angles and torsion angles; correlations between e.s.d.'s in cell parameters are only used when they are defined by crystal symmetry. An approximate (isotropic) treatment of cell e.s.d.'s is used for estimating e.s.d.'s involving l.s. planes.

### Fractional atomic coordinates and isotropic or equivalent isotropic displacement parameters (Å^2^)

**Table:**

	*x*	*y*	*z*	*U*_iso_*/*U*_eq_	
Si1	0.0000	0.80602 (11)	0.2500	0.0536 (4)	
N1	0.5087 (4)	0.4553 (2)	0.40351 (9)	0.0610 (8)	
N2	0.5050 (4)	0.3688 (2)	0.43454 (10)	0.0634 (8)	
N3	0.3420 (5)	0.3270 (3)	0.44279 (12)	0.0804 (10)	
N4	0.2291 (4)	0.3870 (3)	0.41648 (11)	0.0748 (9)	
C1	0.1069 (4)	0.7079 (3)	0.29478 (10)	0.0473 (7)	
C2	0.2904 (5)	0.7098 (3)	0.30746 (12)	0.0600 (9)	
H2	0.3668	0.7655	0.2942	0.072*	
C3	0.3639 (5)	0.6321 (3)	0.33902 (12)	0.0604 (9)	
H3	0.4883	0.6361	0.3465	0.072*	
C4	0.2560 (4)	0.5490 (3)	0.35956 (10)	0.0489 (8)	
C5	0.0720 (5)	0.5455 (3)	0.34793 (12)	0.0679 (10)	
H5	−0.0042	0.4903	0.3617	0.082*	
C6	0.0004 (5)	0.6231 (3)	0.31617 (12)	0.0663 (10)	
H6	−0.1239	0.6186	0.3087	0.080*	
C7	0.3320 (5)	0.4640 (3)	0.39285 (10)	0.0512 (8)	
C8	0.6736 (6)	0.3243 (3)	0.45759 (14)	0.0759 (11)	
H8	0.6317	0.2726	0.4828	0.091*	
C9	0.7768 (6)	0.4185 (4)	0.48099 (16)	0.0952 (15)	
H9A	0.8732	0.3859	0.4999	0.143*	
H9B	0.6949	0.4622	0.5007	0.143*	
H9C	0.8292	0.4687	0.4576	0.143*	
C10	0.7788 (6)	0.2512 (4)	0.42484 (17)	0.1005 (15)	
H10A	0.6997	0.1925	0.4120	0.151*	
H10B	0.8788	0.2151	0.4413	0.151*	
H10C	0.8264	0.2982	0.3998	0.151*	
C11	0.1814 (6)	0.8966 (3)	0.22270 (15)	0.0823 (13)	
H11A	0.2715	0.8474	0.2082	0.123*	
H11B	0.2394	0.9431	0.2464	0.123*	
H11C	0.1273	0.9461	0.1994	0.123*	

### Atomic displacement parameters (Å^2^)

**Table:**

	*U*^11^	*U*^22^	*U*^33^	*U*^12^	*U*^13^	*U*^23^
Si1	0.0603 (8)	0.0454 (7)	0.0552 (7)	0.000	−0.0171 (6)	0.000
N1	0.0659 (18)	0.0600 (18)	0.0569 (16)	0.0010 (15)	−0.0129 (14)	0.0044 (14)
N2	0.081 (2)	0.0553 (17)	0.0538 (16)	0.0102 (16)	−0.0108 (16)	0.0096 (13)
N3	0.084 (2)	0.080 (2)	0.078 (2)	−0.009 (2)	−0.0036 (19)	0.0279 (18)
N4	0.073 (2)	0.080 (2)	0.071 (2)	−0.0060 (17)	−0.0105 (17)	0.0253 (18)
C1	0.0533 (18)	0.0454 (17)	0.0431 (15)	−0.0011 (14)	−0.0056 (14)	−0.0048 (14)
C2	0.055 (2)	0.054 (2)	0.071 (2)	−0.0136 (16)	−0.0133 (17)	0.0106 (17)
C3	0.0481 (19)	0.063 (2)	0.070 (2)	−0.0021 (17)	−0.0174 (16)	0.0072 (18)
C4	0.0549 (18)	0.0506 (18)	0.0413 (15)	−0.0002 (14)	−0.0045 (14)	−0.0031 (14)
C5	0.058 (2)	0.079 (3)	0.066 (2)	−0.0155 (18)	−0.0068 (18)	0.021 (2)
C6	0.0496 (19)	0.084 (3)	0.065 (2)	−0.0095 (19)	−0.0155 (17)	0.0157 (19)
C7	0.061 (2)	0.0526 (19)	0.0403 (15)	0.0018 (15)	−0.0083 (15)	−0.0028 (15)
C8	0.090 (3)	0.071 (3)	0.067 (2)	0.014 (2)	−0.018 (2)	0.012 (2)
C9	0.105 (3)	0.078 (3)	0.103 (3)	0.013 (3)	−0.047 (3)	−0.014 (3)
C10	0.089 (3)	0.094 (3)	0.119 (4)	0.026 (3)	−0.008 (3)	−0.022 (3)
C11	0.088 (3)	0.073 (3)	0.085 (3)	−0.024 (2)	−0.025 (2)	0.028 (2)

### Geometric parameters (Å, °)

**Table:**

Si1—C11	1.853 (4)	C4—C7	1.471 (4)
Si1—C11^i^	1.853 (4)	C5—C6	1.374 (5)
Si1—C1^i^	1.873 (3)	C5—H5	0.9300
Si1—C1	1.873 (3)	C6—H6	0.9300
N1—C7	1.324 (4)	C8—C10	1.471 (6)
N1—N2	1.332 (4)	C8—C9	1.479 (5)
N2—N3	1.301 (4)	C8—H8	0.9800
N2—C8	1.483 (4)	C9—H9A	0.9600
N3—N4	1.309 (4)	C9—H9B	0.9600
N4—C7	1.342 (4)	C9—H9C	0.9600
C1—C2	1.382 (4)	C10—H10A	0.9600
C1—C6	1.388 (4)	C10—H10B	0.9600
C2—C3	1.377 (4)	C10—H10C	0.9600
C2—H2	0.9300	C11—H11A	0.9600
C3—C4	1.370 (4)	C11—H11B	0.9600
C3—H3	0.9300	C11—H11C	0.9600
C4—C5	1.379 (4)		
			
C11—Si1—C11^i^	111.4 (3)	C1—C6—H6	118.8
C11—Si1—C1^i^	110.56 (16)	N1—C7—N4	112.1 (3)
C11^i^—Si1—C1^i^	109.28 (16)	N1—C7—C4	124.2 (3)
C11—Si1—C1	109.28 (16)	N4—C7—C4	123.6 (3)
C11^i^—Si1—C1	110.56 (16)	C10—C8—C9	116.3 (4)
C1^i^—Si1—C1	105.63 (19)	C10—C8—N2	110.4 (3)
C7—N1—N2	100.9 (3)	C9—C8—N2	111.3 (3)
N3—N2—N1	114.6 (3)	C10—C8—H8	106.0
N3—N2—C8	123.1 (3)	C9—C8—H8	106.0
N1—N2—C8	122.4 (3)	N2—C8—H8	106.0
N2—N3—N4	105.8 (3)	C8—C9—H9A	109.5
N3—N4—C7	106.6 (3)	C8—C9—H9B	109.5
C2—C1—C6	115.8 (3)	H9A—C9—H9B	109.5
C2—C1—Si1	124.7 (2)	C8—C9—H9C	109.5
C6—C1—Si1	119.5 (2)	H9A—C9—H9C	109.5
C3—C2—C1	122.4 (3)	H9B—C9—H9C	109.5
C3—C2—H2	118.8	C8—C10—H10A	109.5
C1—C2—H2	118.8	C8—C10—H10B	109.5
C4—C3—C2	120.7 (3)	H10A—C10—H10B	109.5
C4—C3—H3	119.6	C8—C10—H10C	109.5
C2—C3—H3	119.6	H10A—C10—H10C	109.5
C3—C4—C5	118.3 (3)	H10B—C10—H10C	109.5
C3—C4—C7	121.7 (3)	Si1—C11—H11A	109.5
C5—C4—C7	120.0 (3)	Si1—C11—H11B	109.5
C6—C5—C4	120.4 (3)	H11A—C11—H11B	109.5
C6—C5—H5	119.8	Si1—C11—H11C	109.5
C4—C5—H5	119.8	H11A—C11—H11C	109.5
C5—C6—C1	122.4 (3)	H11B—C11—H11C	109.5
C5—C6—H6	118.8		
			
C7—N1—N2—N3	−0.1 (4)	C7—C4—C5—C6	−178.7 (3)
C7—N1—N2—C8	179.8 (3)	C4—C5—C6—C1	−0.5 (6)
N1—N2—N3—N4	0.0 (4)	C2—C1—C6—C5	−0.1 (5)
C8—N2—N3—N4	−180.0 (3)	Si1—C1—C6—C5	177.4 (3)
N2—N3—N4—C7	0.2 (4)	N2—N1—C7—N4	0.2 (4)
C11—Si1—C1—C2	4.3 (3)	N2—N1—C7—C4	179.4 (3)
C11^i^—Si1—C1—C2	−118.6 (3)	N3—N4—C7—N1	−0.3 (4)
C1^i^—Si1—C1—C2	123.2 (3)	N3—N4—C7—C4	−179.4 (3)
C11—Si1—C1—C6	−173.0 (3)	C3—C4—C7—N1	−4.4 (5)
C11^i^—Si1—C1—C6	64.1 (3)	C5—C4—C7—N1	174.9 (3)
C1^i^—Si1—C1—C6	−54.0 (2)	C3—C4—C7—N4	174.7 (3)
C6—C1—C2—C3	0.5 (5)	C5—C4—C7—N4	−6.0 (5)
Si1—C1—C2—C3	−176.9 (3)	N3—N2—C8—C10	103.6 (4)
C1—C2—C3—C4	−0.3 (5)	N1—N2—C8—C10	−76.3 (5)
C2—C3—C4—C5	−0.2 (5)	N3—N2—C8—C9	−125.6 (4)
C2—C3—C4—C7	179.1 (3)	N1—N2—C8—C9	54.4 (5)
C3—C4—C5—C6	0.6 (5)		

Symmetry codes: (i) −*x*, *y*, −*z*+1/2.

### Hydrogen-bond geometry (Å, °)

**Table:**

Cg is the centroid of the tetrazole ring.

**Table:**

*D*—H···*A*	*D*—H	H···*A*	*D*···*A*	*D*—H···*A*
C9—H9B···Cg^ii^	0.96	2.86	3.738 (5)	152

Symmetry codes: (ii) −*x*+1, −*y*+1, −*z*+1.
